# Non-Isothermal Dynamic Mechanical Analysis of Ribbon Metallic Glasses and Its Thermodynamic Description

**DOI:** 10.3390/ma15238659

**Published:** 2022-12-05

**Authors:** Arseniy Berezner, Victor Fedorov

**Affiliations:** Theoretical and Experimental Physics Department, Derzhavin Tambov State University, Internatsionalnaya str. 33, 392000 Tambov, Russia

**Keywords:** metals and alloys, cyclic deformation, external energy influences

## Abstract

In this work, derivation of the main thermodynamic relationships is realized together with the applied calculation of some parameters, providing the systematized description of non-linear thermo-mechanical deformation at dynamic mechanical analysis (DMA). Obtained equations and values agree well with experiments on different ribbon metallic glasses. We generalize the main initial conditions (i.e., experimental and numerical parameters) by that the proposed model can be used for the investigation of DMA in different materials. The further opportunities of the found approach are also discussed in frames of phase transitions in metallic glass.

## 1. Introduction

Formally, the study of deformation in materials has been developing in physics and technics since the forming of different scientific fields [1]. After comprehensive investigations of the elastic response in materials [2], scientists observed isothermal [3] or non-isothermal [4] creep and internal friction [5]. The investigated materials varied from metals [6] or simple inorganic compositions [7] to complex polymer systems [8]. Some deformation features (for example, yield drop, non-linear plastic stage and so on) appearing at elasto-plastic deformation also are described in frames of the different structural models [9]. Viscoelastic deformation is quite well described both in continuum mechanics [10] and by the semi-empirical models [11]. However, non-isothermal conditions (with different regimes of mechanical loading) are not properly investigated due to the absence of a general model describing the temporal or temperature deformation function. One of them is in material response to variable (uniform in time) heating with periodic force impact (dynamic mechanical analysis, i.e., DMA) [12]. The study of this deformation case by model generalization is of interest in physics and technics not only from fundamental but also from applied standpoints.

Along with corrosion [13] and magnetic [14] properties, mechanical parameters of different amorphous alloys (AMA) have been intensively investigated [15] since the first synthesis of metallic glass (MG) [16]. As these materials have some quantitative advantage by the mentioned properties, analysis of their behavior is more actual for the industry [17]. Viscosity [18] and dynamic properties [19] are intensively investigated in MG under different conditions (such as annealing [20] and rolling [21]), but mainly elastic (Kelvin–Voigt) deformation stage is the most investigated yet. Traditionally, an analogy is drawn between amorphous alloys, polymers and liquids [22] because the interatomic disordered structure is typical for these materials and substances [23]. However, the differences between their chemical composition and state must be accounted, and that is not always taking place because of insufficient data on this matter. For example, the Kauzmann paradox [24] is proposed to exist for metallic glasses [25] but not for all cases of their deformation, the analytical view of entropy function and other parameters, mentioned in the original work (i.e., in [24]), is known. Thus, taking into account described points and other ones, thermodynamic description (with applied calculations) of cyclic inelastic deformation (DMA) of as-prepared and rolled MG is the main goal of this work.

## 2. Materials and Methods

As a basement for this investigation, our previous experimental data on DMA of Al- and Cu-based ribbon amorphous alloys (with ~40 μm of thickness and 4.5 mm of width) were chosen [26,27,28]. The general conditions for DMA are in the presence of *F_load_*. Preloading (constant during an experiment) and oscillating (with *ω* = 6π rad/s frequency and *A* amplitude) force, which impact a specimen together. Moreover, continuous heating of alloy (with *V*_T_ = 5 K/min constant rate) from the heater was carried out in *t* time that led to break of the specimen in the *B* moment. During experiment, each specimen elongates from *l_0_*~18.5 mm of working size (at potentially variable length) up to the critical maximum (~27 mm). Further model analysis must be carried out, starting from our previous equations, which were obtained in [26] for *l* variable deformation and *F_react._* reaction force:

(1) $$l(t)=l_{0}+\frac{Ct}{B^{2}-Bt},$$

(2) $$F_{react.}(\omega ;t(T))=F_{load}-A\sin(\omega t)-\frac{2mC}{{\left(B-t\right)}^{3}}.$$

For uniform temporal heating, from *T*_0_ (~300 K) temperature to variable *T* one (not above ~550 K and 568 K crystallization temperatures of Al-based and Cu-based MG, consequently), we can write the time–temperature relationship:

(3) $$t=\frac{T-T_{0}}{V_{T}}=\frac{\Delta T}{V_{T}}.$$

Moreover, *T* and *T*_0_ are temperatures of the environment near the specimen (the pure temperature of alloy cannot be precisely measured because of technical calorimetric principles). In our experiments, different parasitic thermal effects (such as self-heating) arising at cyclic deformation in other systems [29] are not registered by a pyrometer or thermocouple. For Al- and Cu-based alloys, the mean values, presented in Table 1, are typical, among which *C* is a personal deformation coefficient, and *m* corresponds to the mass of a specimen.

Generally, parameters in Equations (1)–(3) can take different numbers, depending on variable values of DMA. Further model derivations will be realized based on the described Equations (1)–(3) and data from Table 1.

## 3. Results and Discussion

Before solving a thermodynamic problem, the boundaries of investigated system with external and internal parameters [30] are necessary to determine. Thus, let us consider the «load-specimen» or «machine grip-specimen» system, characterized by *a* = *l* (length) external parameter and *T* temperature, and that is heated with *δQ* amount (of heat) for change its *dU* internal energy with further doing of *δW* work on its environment (i.e., work of a specimen on the machine grip and force sensors). With respect to the construction features of the DMA machine, its furnace can be considered a container that does not exchange heat with external space. As a generalized force in this system, reaction one (2) is chosen, and its analytical form can be represented as a function both of length and temperature [28]:

(4) $$F_{react.}(l;T)=F_{load}-A\sin\left(\omega \frac{T-T_{0}}{V_{T}}\right)-\frac{2mC}{{\left(B-\frac{B^{2}(l-l_{0})}{C+B(l-l_{0})}\right)}^{2}\left(B-\frac{T-T_{0}}{V_{T}}\right)}.$$

Moreover, preloading force (i.e., *F_load_*) is already accounted for the description of the initial system state, and, therefore, the elastic (post-preloading) transition process in a specimen is not necessary for consideration at DMA. Such delimitation in the system permits optimal modeling of the oscillating force as an inner process instead as a part of the external work performed on the specimen. This feature is also justified by the anharmonic amplitude-frequency response of the specimen (due to the change of the *ω*(*T*) in the sinus argument of Equation (2) or (4)) in some range at further temperature growth [26,28]. Thus, the volume of the thermodynamic system is set some larger than the personal sizes of a specimen, but all its parts preserve or change reversibly their properties, unlike alloy, which deforms.

For a more valid model derivation and further analysis of DMA, the mentioned Equation (4) also can be checked with thermodynamic criteria of a state function. It is possible to do this with generalized force (4) by the equation of a differential form [31]. In this case, equality of the mixed partial derivatives ${\left(\frac{\partial^{2}F_{react.}(l;T)}{\partial l\partial T}\right)}_{T}={\left(\frac{\partial^{2}F_{react.}(l;T)}{\partial T\partial l}\right)}_{l}$ takes place, and both parts are equal to $-\frac{4mB^{2}C^{2}}{V_{T}{\left(B-\frac{T-T_{0}}{V_{T}}\right)}^{5}{\left(C+B(l-l_{0})\right)}^{2}}$. On the whole Σ(*l*;*T*) definition domain and trajectory *l*(*T*), i.e., on a deformation curve (process), the function (4) is continuous. Therefore, proposed equations can be considered for a deterministic description of deformation because *F_react._* value is the state function, described by an equation of state, i.e., by Equation (4), on the Σ(*l*;*T*) connected domain. Note that, because of the metastable initial state, the deformation of metallic glass can be considered in frames of equilibrium thermodynamics with a possible change of the obtained equations onto inequalities at non-equilibrium conditions (for example, near the crystallization temperature). As the thermal equation of state is known, the caloric one, i.e., full internal energy, can be found. From the first thermodynamic law *δQ* = *dU* + *δW*, full energy Δ*U* will be determined as the difference between whole heat Δ*Q* and work Δ*W*. Moreover, this law (equation) is integrated with respect to the deformation process (L(*l*(*T*);*T*) with a non-linear trajectory), taking into account Equation (4), i.e., as $\int_{L}\delta Q=\int_{L}dU+\int_{L}\delta W=\int_{L}dU+\int_{L}F_{react.}(l;T)dl$. We also notice that curvilinear integral over *δQ* must be conditionally equalized to the full amount of heat received from an electric heater in a furnace (by Joule’s law)

(5) $$\Delta Q=Pt=P\frac{\Delta T}{V_{T}}$$

where *P* is electrical heater power and *t* is heating duration according to Equation (3). This condition can give some overvalue number for real heating of alloy because of secondary heat transfer from the thermal element to inner furnace walls and environment of a specimen. However, this value has a fixed limit, not exceeding all generated heat (5), and it permits estimation of the system dynamics up to the constant difference. Actually, the amount of heat, passing only through the specimen, could be calculated with the different thermal models (such as Fourier or Maxwell–Cattaneo laws) instead of Equation (5), but the real heat flow dynamic and its spatial distribution in the alloy are hard to estimation from the experimental standpoint. Therefore, we use standard full estimation (5) for analysis. After curvilinear integration of *δW*, an equation for work at DMA of MG has a form:

(6) $$\Delta W=\frac{F_{load}C\left(T-T_{0}\right)-AC\Theta \left(T-T_{0}\right)}{BV_{T}\left(B-\frac{T-T_{0}}{V_{T}}\right)}+\frac{C^{2}m\left({\left(B-\frac{T-T_{0}}{V_{T}}\right)}^{4}-B^{4}\right)}{2B^{4}{\left(B-\frac{T-T_{0}}{V_{T}}\right)}^{4}},$$

where Θ (|Θ|≤1) is a multiplier of $\int_{T_{0}}^{T}\frac{AC\sin\left(\frac{\omega (T-T_{0})}{V_{T}}\right)}{V_{T}{\left(B-\frac{T-T_{0}}{V_{T}}\right)}^{2}}dT$, according to the mean value theorem [31], which takes place at curvilinear integration, then, full internal energy will be determined as:

(7) $$\Delta U=\Delta Q-\Delta W=P\frac{\Delta T}{V_{T}}-\frac{(F_{load}C-AC\Theta )\left(T-T_{0}\right)}{BV_{T}\left(B-\frac{T-T_{0}}{V_{T}}\right)}-\frac{C^{2}m\left({\left(B-\frac{T-T_{0}}{V_{T}}\right)}^{4}-B^{4}\right)}{2B^{4}{\left(B-\frac{T-T_{0}}{V_{T}}\right)}^{4}}.$$

**Figure 1:**
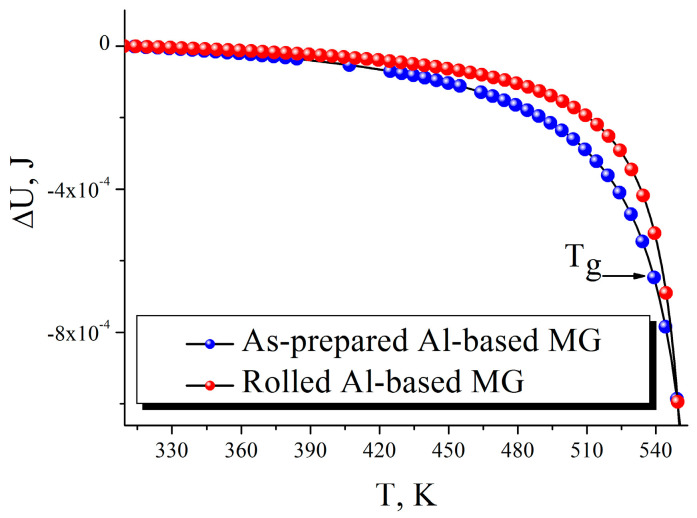
Change of full internal energy in Al_85_Y_8_Ni_5_Co_2_ MG with rolling (red dots) or without it (blue dots). The glass-transition temperature (*T_g_*) is mentioned with the arrow.

Analysis of Equation (6) shows that work conducted in the considered system is positive that corresponds to the action of the deformation force, directed along a total shift of the machine grip. Up to the term of supplied heat, the full internal energy of the specimen decreases during deformation (see Figure 1).

The calculated curves in Figure 1 correlate with our hypothesis (in the collective work [28]) about structural rearrangement to a more uniform change of full internal energy after rolling. It also agrees with our interpretation that rolled and as-prepared specimens achieve joint minimal energy points near *T_g_* (~540 K) [28]. Amplitude-frequency and phase-frequency modulations, arising at the deformation of amorphous alloys [26,27,28], do not sufficiently impact the main decrease tendency of function (7), but some variation for its rate takes place. Change of *C* and *B* parameters corresponds quantitatively with the thermodynamic functions (4)–(7) that also testifies about the strong relationship between these values and the deformation conditions of the material. Moreover, by Equation (7), we obtain the same tendency for Cu-based alloy (decrease of Δ*U*(*T*) up to the minimum), and it changes faster near personal *B* time (2827 s) than for Al-based one. The difference in energy dynamics of both alloys (if we compare their *B* parameters from Table 1) causes a relatively earlier fracture of the Cu-based ribbon. However, any comparison of these alloys in an amorphous state is limited by *T_g_* of copper-based MG (528 K) [27].

In some works on metallic glass thematic (such as described in the review [32]), the structural condition is described in frames of energy Arrhenius model, i.e., by Maxwell–Boltzmann (generally, Gauss) statistics. Moreover, this approach is often supposed to be applicable for the glass transition region. However, by definition [33], the mentioned statistics take place in the equilibrium state with a predominantly fixed temperature that obstructs the model using in more complex cases (for example, in a non-isothermal process with loading or at a phase transform point). Someone can note the necessity in a more complex modified exponential function at a strong behavioral deviation of alloy from ideal normal distribution [34]. These features make necessary a thermodynamic estimation of statistics in the conditions of DMA.

By opening the brackets in Equation (7) with further term division of the numerator by the denominator, full internal energy can be represented in a form:

(8) $$\Delta U=P\frac{\Delta T}{V_{T}}-\frac{F_{load}C}{B-\frac{T-T_{0}}{V_{T}}}+\frac{F_{load}C}{B}+\frac{AC\Theta }{B-\frac{T-T_{0}}{V_{T}}}-\frac{AC\Theta }{B}+\frac{C^{2}m}{2{\left(B-\frac{T-T_{0}}{V_{T}}\right)}^{4}}-\frac{C^{2}m}{2B^{4}}.$$

Then, the remembered expression of 〈U(T)〉 mean (i.e., expected) energy by its distribution (and probability density function, i.e., PDF) [33], we can write the equations:

(9a) $$\Delta U\left(T_{1/2}\right)∼\langle U\left(T\right)\rangle =\int_{T}U\left(T\right)f\left(T\right)dT;$$

(9b) $$\begin{array}{l} \frac{d\langle U(T)\rangle }{dT}=U(T)f(T)\sim \frac{d(\Delta U(T_{1/2}))}{dT}=\\ =\frac{P}{V_{T}}-\frac{F_{load}C}{V_{T}{\left(B-\frac{T_{1/2}-T_{0}}{V_{T}}\right)}^{2}}+\frac{AC\Theta }{V_{T}{\left(B-\frac{T_{1/2}-T_{0}}{V_{T}}\right)}^{2}}+\frac{2C^{2}m}{V_{T}{\left(B-\frac{T_{1/2}-T_{0}}{V_{T}}\right)}^{5}} \end{array},$$

where *T*_1/2_ is the mean experimental temperature (~430 K), *ΔU*(*T*_1/2_) is the function value of (8) at the middle-temperature point, *f*(*T*) is a derivative of the distribution function (in the differential *dP*(*T*) = *P′*(*T*)*dT* = *f*(*T*)*dT*, i.e., PDF. *T*_1/2_ can be considered as a variable if we describe the whole experimental data array, i.e., variation of the experimental mean point is taken into account for different DMA tests occurring for the same alloy system (specimen set) in frames of Equation (8). In the second term of (9b), we note the $U_{1}=\frac{F_{load}C}{B-\frac{T_{1/2}-T_{0}}{V_{T}}}$ multiplier, also being in (8), and write the recurrent equation (that takes place not only in *T*_1/2_ point but at different *T*):

(10) $$\frac{dU_{1}}{dT}=\frac{U_{1}}{V_{T}\left(B-\frac{T-T_{0}}{V_{T}}\right)},$$

which is a differential one with separated variables, and its general solution (i.e., *U*_1_) can be represented in a form:

(11a) $$U_{1}=M_{1}\exp\left(\int \frac{dT}{V_{T}\left(B-\frac{T-T_{0}}{V_{T}}\right)}\right)$$

where *M*_1_ is an integration constant. Performing similar steps in the third and fourth terms in (9b), let us derive equations:

(11b) $$U_{2}=M_{2}\exp\left(\int \frac{dT}{V_{T}\left(B-\frac{T-T_{0}}{V_{T}}\right)}\right),\;U_{3}=M_{3}\exp\left(\int \frac{4dT}{V_{T}\left(B-\frac{T-T_{0}}{V_{T}}\right)}\right)$$

in which *M*_2_ and *M*_3_ are integration constants of the corresponding differential equations. By substitution of (11a) and (11b) in the left part of (9a) instead *U*_1_, *U*_2_ and *U*_3_ with *M*_0_ notation for a sum of all remained terms, we find the relationship:

(12) $$M_{0}+M_{1}\exp\left(\tau \right)+M_{2}\exp\left(\tau \right)+M_{3}\exp\left(4\tau \right)\sim \langle U(T)\rangle =\int_{T}U(T)f(T)dT,$$

where $\tau =\int \frac{dT}{V_{T}\left(B-\frac{T-T_{0}}{V_{T}}\right)}$. Using the mean value theorem for the integral in (12), let us derive the expression:

(13) $$M_{0}+M_{1}\exp\left(\tau \right)+M_{2}\exp\left(\tau \right)+M_{3}\exp\left(4\tau \right)\sim \langle U(T)\rangle =\bar{u}\int_{T}f(T)dT$$

where both parts can be divided by $\bar{u}$ (i.e., mean value of *U*(*T*) at *T*~*T*_1/2_), and after differentiation by *T*, the final estimation form for PDF will be found as:

(14) $$\alpha_{0}+\alpha_{1}\exp\left(\tau \right)+\alpha_{2}\exp\left(\tau \right)+\alpha_{3}\exp\left(4\tau \right)\sim f(T)$$

with *α_k_* exponential coefficients (i.e., the quotients of $M_{k}$/$\bar{u}$, (*k* = 0–3)).

As seen from (14), *f*(*T*) can be described by the sum of exponents, and its normalizing condition $\int_{T}f(T)dT=1$ will be determined by the equality between $\bar{u}$ and the sum in the left part of (13), i.e., the precise equation between 〈U(T)〉 and $\Delta U(T_{1/2})$ is potentially possible at *T = T*_1/2_ (or description will be qualitative at *T~T*_1/2_). Moreover, the number of terms (depending on *M_k_*) and value of *τ* argument in exponents provide the view of *f*(*T*) in each experiment. This variation of the coefficients can lead to quite different functional representations, which have a similar curve form but not the same interpolation accuracy for the experiment. Integrals in exponent arguments (i.e., in Equation (11a,b)) also can be considered in frames of mean value theorem that gives $\sim \exp\left(\frac{\Delta T}{V_{T}(B-\Delta T_{1/2}V_{T}{}^{-1})}\right)$ an expression for them. Moreover, by exact integration in *τ*, $\sim \frac{1}{B-\Delta T_{1/2}V_{T}{}^{-1}}$ expression is an alternative form for the same equations with analogous functional behavior, compared with exponents. The existence of many analytical representations for *f*(*T*) (also at the numerical fitting of experiments [32]) can be caused by a change of interaction behavior between atoms and molecules in metallic glass. Arrhenius equation is typical for more ideal equilibrium systems with independent «particles» interacting elastically with one another [33]. The presence of non-elastic pair interactions between «particles» leads to a change of PDF and related values, which can be observed in Debye’s and Einstein’s works on heat capacity or in Van der Waals’s article, for example [35]. During DMA of amorphous alloys with heating above room temperatures, probable anharmonic interaction of the atoms and molecules is similar to simultaneous periodical stretching of many elastic and non-elastic springs, acting between material points. In this case, the collective resonance effects with specific experimental signals (relaxation maxima, beating and so on) are possible.

**Figure 2:**
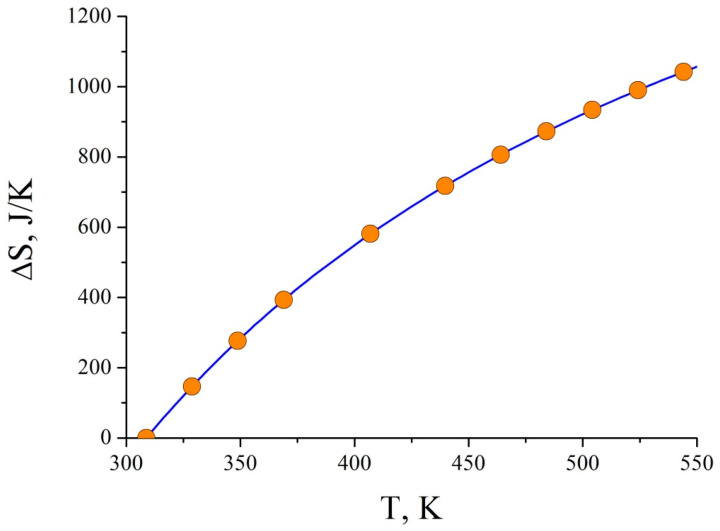
Entropy change at DMA of Al-based and Cu-based amorphous alloys.

In frames of applied thermodynamics, molar capacity and thermal expansion coefficient are often determined. Moreover, the estimation of entropy change in a process is an important task. As we derive the main functions of the state, an analysis of the described parameters can be provided. Particularly, from Equation (5), the average entropy change function has a form:

(15) $$\Delta S=\frac{\Delta Q}{T}=P\frac{\Delta T}{TV_{T}},$$

and its plot is depicted in Figure 2.

As seen from Figure 2, non-isothermal deformation with mechanical loading is in equilibrium only at room temperature (initial conditions) because Δ*S* = 0. The further temperature growth leads to the development of a non-equilibrium process (Δ*S* > 0). Therefore, using only equilibrium models (such as Maxwell–Boltzmann statistics) appears to be ineffective in the high-temperature range. Moreover, from Equation (15), we can notice that temperature impact is the main source of the entropy change, i.e., thermal activation is a distinctive feature for this type of experiment.

Previously [36], we calculated linear thermal expansion coefficient (CLTE), whose number value agreed with the experiment up to the integer part of a number and magnitude order (α_L_~4·10^−6^–6·10^−6^ 1/K for Cu-based and Al-based MG, consequently). It permits estimation of molar heat capacity, which relates with CLTE linearly by *γ* Gruneisen parameter [37] for many materials. In most cases, precise analytical derivation of *γ* is complicated by different factors, but this proportion coefficient can be specified empirically. As known [38], the thermal capacity of amorphous alloys varies less intensively, unlike partially crystallized (i.e., post-annealed) one, near *T_g_*. However, the estimation of this value by «crystalline» data gives a confidential interval. From the literature [39], we observe that the ratio between molar heat capacity *C_mol_* and CLTE (i.e., *b* = *C_mol_*/α_L_) changes on 15–30 units for most crystalline alloys, and, therefore, *C_mol_* for Al- and Cu-based metallic glasses lies between the numbers α_L_·*b =* 60–180 J/(mol·K). This result agrees with experimental data by the magnitude order both for crystalline and amorphous alloys [40,41]. Some our model relationships can be considered as a basement for analysis of the different systems or conditions, such as complex deformation of Al-based [42] or Ti-based alloys [43].

## 4. Conclusions

In this work, we carried out consistent calculations of the main thermodynamic relations for Al- and Cu-based amorphous alloys, which underwent acting of uniform (in time) heating and oscillating mechanical load. Non-equilibrium deformation behavior, which limits using of Maxwell–Boltzmann statistics in a wide high-temperature interval (above room ones), has been shown. CLTE and molar heat capacity, which are sensitive to molecular rearrangements, are calculated in frames of the proposed model. Moreover, our model corresponds quite precisely with experimental data from the different works on amorphous and crystalline thematics. Derived relationships can be used to analyze DMA in different materials that demonstrate the same behavior in similar conditions. Using the thermal and caloric state equations permits analysis of cyclic processes and system stability (by potentials) that can give additional information about different properties of the investigated systems near phase transition points.

## Figures and Tables

**Table 1:** Experimental parameters and personal material values of investigated MG.

Alloy (at.%)	*C*, [m·s]	*B*, [s]	*m*, [kg]	*F_load_*, [N]	*A*, [N]
Al_85_Y_8_Ni_5_Co_2_	0.078	3157	10^−4^	3.6	0.0036
Cu_54_Pd_28_P_18_	0.0492	2827	10^−4^	0.9	0.0063

## Data Availability

Not applicable.
